# Uncoupling protein 2 gene polymorphisms are associated with obesity

**DOI:** 10.1186/1475-2840-11-41

**Published:** 2012-04-25

**Authors:** Sukma Oktavianthi, Hidayat Trimarsanto, Clarissa A Febinia, Ketut Suastika, Made R Saraswati, Pande Dwipayana, Wibowo Arindrarto, Herawati Sudoyo, Safarina G Malik

**Affiliations:** 1 Eijkman Institute for Molecular Biology, Jl. Diponegoro 69, Jakarta, 10430, Indonesia; 2 Division of Endocrinology and Metabolism, Department of Internal Medicine, Faculty of Medicine Udayana University, Jl. P.B. Sudirman and Sanglah Hospital, Jl. Kesehatan, Denpasar, Indonesia

**Keywords:** UCP2, Ala55Val, G(−866)A, Obesity, Serum lipids, Type 2 diabetes mellitus

## Abstract

**Background:**

Uncoupling protein 2 (UCP2) gene polymorphisms have been reported as genetic risk factors for obesity and type 2 diabetes mellitus (T2DM). We examined the association of commonly observed UCP2 G(−866)A (rs659366) and Ala55Val (C > T) (rs660339) single nucleotide polymorphisms (SNPs) with obesity, high fasting plasma glucose, and serum lipids in a Balinese population.

**Methods:**

A total of 603 participants (278 urban and 325 rural subjects) were recruited from Bali Island, Indonesia. Fasting plasma glucose (FPG), triglyceride (TG), high density lipoprotein cholesterol (HDL-C), low density lipoprotein cholesterol (LDL-C) and total cholesterol (TC) were measured. Obesity was determined based on WHO classifications for adult Asians. Participants were genotyped for G(−866)A and Ala55Val polymorphisms of the UCP2 gene.

**Results:**

Obesity prevalence was higher in urban subjects (51%) as compared to rural subjects (23%). The genotype, minor allele (MAF), and heterozygosity frequencies were similar between urban and rural subjects for both SNPs. All genotype frequencies were in Hardy-Weinberg equilibrium. A combined analysis of genotypes and environment revealed that the urban subjects carrying the A/A genotype of the G(−866)A SNP have higher BMI than the rural subjects with the same genotype. Since the two SNPs showed strong linkage disequilibrium (D’ = 0.946, r^2^ = 0.657), a haplotype analysis was performed. We found that the AT haplotype was associated with high BMI only when the urban environment was taken into account.

**Conclusions:**

We have demonstrated the importance of environmental settings in studying the influence of the common UCP2 gene polymorphisms in the development of obesity in a Balinese population.

## Background

Obesity and type 2 diabetes mellitus (T2DM) have become global health concerns. The International Obesity Task Force estimated that approximately one billion adults are overweight, and 475 million of them are obese [1]. In 2010, it was predicted that 285 million people might suffer from T2DM worldwide [2]. Obesity and T2DM would eventually develop into metabolic disorders and coronary heart disease (CHD), in particular when accompanied with a high serum lipid level. The rapidly increasing prevalence of obesity and T2DM, as well as a high serum lipid level were markedly noticed in developing countries, proposed to be the result of adopting a Western lifestyle, and characterized by increased consumption of energy-dense food and reduced physical activity [3,4].

Obesity and T2DM manifested from a combination of genetic predispositions and unfavorable environmental factors, involving unbalanced energy intake and expenditures for basal metabolic process and physical activity [5,6]. One of the key regulators of energy balance is the uncoupling protein 2 (UCP2), a transporter protein present in the mitochondrial inner membrane (UniProt accession P55851) [7]. UCP2 mediates proton leak across the inner membrane via uncoupling of the substrate oxidation from ATP synthesis, thus decreasing ATP production by mitochondrial respiratory chain. Consequently, UCP2 activity may suppress glucose-stimulated insulin secretion, which is regulated by the ATP/ADP ratio [8,9]. As shown in animal models and human islets, over-expression of UCP2 was associated with reduced insulin secretion [10,11]. The effect of UCP2 on insulin secretion has been reported to extend towards regulation of fat metabolism, either directly or indirectly [12], by promoting fat oxidation [13]. In response to fasting, UCP2 was reported to promote degradation of triacylglycerols [14] and fatty acid oxidation by limiting glycolysis [15]. Other reports showed that UCP2 expression appeared to be up-regulated in response to a high fat diet [12,16,17]. Taken together, these reports suggest that UCP2 has a role in counteracting diet-induced obesity by enhancing peripheral fatty acid metabolism.

The commonly observed UCP2 gene single nucleotide polymorphisms (SNPs) G(−866)A in the promoter region (rs659366) and Ala55Val (a C to T substitution) in exon 4 (rs660339) have been associated with obesity and T2DM to a certain degree. The G(−866)A has been shown to affect the UCP2 expression level, since this mutation is located within the *cis* regulatory region that interacts with multiple nuclear *trans* activating factors [18]. The minor allele was correlated with reduced obesity risk [19]. In various ethnicities, the UCP2 G(−866)A SNP has also been associated with central obesity [20], hyperinsulinemia in obese subjects [21], and increased or decreased risk of CHD [22-24]. Other reports have associated the A allele with increased levels of HDL-C and decreased levels of LDL-C in a Chinese population [25], and reduced LDL particle size levels [26]. The 55Val/Val genotype of the Ala55Val polymorphism was reported to be associated with a higher incidence of T2DM in Caucasians and African-Americans [27], similar to our previous study in a limited Balinese urban population [28]. This variant also showed an association with a higher fasting insulin level in obese Taiwanese aborigines [29]. Studies on metabolic rate measurement have found a lower 24-h energy expenditure [30] and a higher efficiency of energy exercises [31] in subjects carrying 55Val/Val. Thus, people with this genotype would have more efficient energy use due to a lower degree of uncoupling, resulting in the increased ATP and reactive oxygen species (ROS) production [8].

In this study, we aim to examine the association of the UCP2 gene G(−866)A and Ala55Val SNPs with obesity, high FPG and serum lipids in a Balinese population, which has not been characterized for the SNPs, comparing the urban and rural population. Apart from our previous report in a limited Balinese urban population [28], there was no other report on the association of these SNPs with obesity, high FPG and serum lipids in the Balinese. Balinese people have been exposed to lifestyle changes brought by tourism development in the last two decades. We propose that the effect of these changes, reflected by the high prevalence of subjects with obesity, high FPG, and serum lipids disorder, was modulated by UCP2 SNPs.

## Materials and methods

### Subjects and study design

A cross-sectional study was conducted, enrolling 603 random participants from Bali, Indonesia, with informed consent (ethical approval was granted from the Faculty of Medicine Ethic Committee, Udayana University, and the Eijkman Institute Research Ethics Commission) [32]. This study was part of the metabolic disorder study done in Bali, where DNA samples were available [33]. The subjects were recruited by random sampling from either urban (278 subjects) or rural (325 subjects) settlements, and then stratified for large villages and sub-villages. Mean of subjects’ age is 46 ± 10 years and 52 ± 16 for urban and rural populations, respectively.

### Measurements

Anthropometric measurements were taken, including body mass index (BMI) which was calculated as body weight in kilograms divided by the square of height in meters. Study subjects were classified as obese (BMI ≥25 kg/m^2^) according to the Asia-Pacific perspective redefining obesity in adult Asian [34]. It has been reported that higher metabolic risks were observed in Asian at a given BMI as compared to Caucasian, proposed to be due to the interplay of genetic susceptibility and environmental factors [35].

Blood samples were drawn after at least 10 hours overnight fasting. Fasting plasma glucose (FPG) was measured using the standard hexokinase method. High FPG of ≥126 mg/dL was determined based on the WHO recommendations [36]. To determine the fasting serum lipids, standard spectrophotometric methods were employed. The examined fasting lipid profiles were triglycerides (TG), high density lipoprotein cholesterol (HDL-C), low density lipoprotein cholesterol (LDL-C) and total cholesterol (TC). Classifications of TG, HDL-C, LDL-C, and TC were based on The National Cholesterol Education Program (NCEP)-Adult Treatment Panel III (ATPIII) guidelines on detection, evaluation, and treatment of high blood cholesterol in adults [37]. Classifications of TG were: normal <150 mg/dL; borderline 150–199 mg/dL; high 200–499 mg/dL; very high ≥500 mg/dL. Classifications of HDL-C were: low <40 mg/dL; normal 40–59 mg/dL; high ≥60 mg/dL. Classifications of LDL-C were: optimal <100 mg/dL; near optimal 100–129 mg/dL; borderline high 130–159 mg/dL; high 160–189 mg/dL; very high ≥ 190 mg/dL. Classifications of TC were: desirable <200 mg/dL; borderline high 200–239 mg/dL; high ≥240 mg/dL [37].

### DNA Extraction and Genotyping

Genomic DNA was extracted from blood spots in Guthrie Cards (Whatman, Clifton, NJ, USA) using a Chelex-100 (Bio-Rad, Hercules, CA, USA) protocol [38], or blood sample in EDTA tubes using a modified salting out method [39]. SNPs detections were performed by polymerase chain reaction-restriction fragment length polymorphisms (PCR-RFLP) method, as described previously by Sesti *et al.* for the G(−866)A polymorphism [40] and Kubota *et al.* for the Ala55Val polymorphism [41]. In brief, a DNA fragment corresponding to the UCP2 G(−866)A polymorphism (rs659366) was amplified by 5’-CACGCTGCTTCTGCCAGGAC-3’ as forward primer and 5’-AGGCGTCAGGAGATGGACCG-3’ as reverse primer [40]. PCR products were digested by *Mlu*I restriction enzyme (NEB, Ipswich, MA, USA) and separated on 2% agarose gel electrophoresis. The (−866)AA genotype was marked by a single 363 bp fragment due to lost of *Mlu*I site, while the wild-type (−866)GG genotype was digested into 295 bp and 68 bp fragments. For the Ala55Val polymorphism (rs660339), a DNA fragment was amplified using a pair of oligonucleotide primer containing mismatched bases: forward primer 5'-CTGGAGTCTCGATGGTGTCTAC-3', and reverse primer 5'-CACCGCGGTACTGGGCGTTG-3' that possessed a *Hin*cII site [41]. The 198 bp PCR products were digested with *Hin*cII (NEB, Ipswich, MA, USA), the Val55Val genotype was marked by the presence of the *Hin*cII site, resulting in two DNA fragments of 179 bp and 19 bp which were subjected to 3% agarose gel electrophoresis (Lonza, Basel, Switzerland).

### Statistical Analyses

The data exploration and analysis was performed by utilizing custom scripts for data preprocessing and R statistical packages employing *genetics*, *rms* and *haplo.stats* libraries. Continuous variables were shown as mean ± SD and compared by performing the Welch’s *t*-test for variables with normal distribution or the Mann–Whitney *U*-test for variables with skewed distribution. Departure of the genotype distribution to Hardy-Weinberg equilibrium was determined using the Fisher’s exact test. Linkage disequilibrium was measured using *D’* and *r*^*2*^ statistics as implemented in the *genetics* library. We considered *p*-values of less than 0.05 as significant. The *p*-values for genotype frequency of each SNP between urban and rural population were calculated using Fisher’s exact test on 2 × 3 contingency table.

In order to determine the association between UCP2 genotypes and disease traits, multiple ordinal logistic regressions were conducted with adjustment for age, gender and environment (urban vs. rural). The logistic regressions were performed as implemented in the *rms* library from R statistical packages. All disease traits were categorized as previously defined, and the associated *p*-values were calculated using the Wald test.

The association between UCP2 haplotypes and disease traits were determined by logistic regressions which employed the expectation maximization (EM) algorithm for determining the haplotypes as implemented in **haplo.glm** function of *haplo.stats* library from R statistical packages. Both the additive and dominant models of genetic effects were considered for the analysis, with adjustments for age, gender and population. The recessive model was not feasible for an analysis due to a very low frequency of predicted samples that possesses the rare recessive haplotype. The use of logistic regressions using the expectation maximization (EM) algorithm was described in detail elsewhere [42-44].

## Results

### Baseline characteristics

The baseline characteristics of study subjects (278 urban and 325 rural Balinese) were listed in Table 1. The overall prevalence of obesity (BMI > 25 kg/m^2^) was 51% in urban (58% in male, 40% in female), and 23% in rural (24% in male, 22% in female) subjects. In the non-obese group, the urban subjects (both male and female) showed significantly higher BMI as compared to the rural subjects (all *p* < 0.001). The rural females have higher FPG level compared to the urban females (*p* = 0.005), although the level was much below the hyperglycemia limit of 126 mg/dL. In the obese group, the rural Balinese showed higher TC level than their urban counterparts (*p* = 0.025), specifically in males (*p* = 0.039).

**Table 1:** Baseline characteristics of study subjects

**Variable**	**Non-Obese**			**Obese**		
	**Urban**	**Rural**	***p***	**Urban**	**Rural**	***p***
*All, n*	*136*	*250*		*142*	*75*	
Age	46.0 ± 10.9	52.3 ± 16.8	**< 0.001**	46.3 ± 9.3	49.1 ± 11.5	0.073
FPG	95.2 ± 25.2	97.1 ± 18.9	0.426	99.9 ± 43.7	94.9 ± 11.8	0.204
BMI	22.4 ± 1.9	20.7 ± 2.6	**< 0.001**	28.6 ± 3.5	27.9 ± 2.1	0.063
TG^α^	126.4 ± 63.7	118.5 ± 53.8	0.344	160.1 ± 74.6	156.6 ± 69.8	0.882
HDL-C^α^	53.2 ± 11.4	54.2 ± 13.9	0.698	48.1 ± 10.6	51.0 ± 9.8	0.054
LDL-C	119.6 ± 34.3	120.6 ± 29.6	0.781	119.4 ± 31.7	126.8 ± 34.9	0.127
TC	198.1 ± 38.2	196.3 ± 34.8	0.638	199.5 ± 36.1	211.0 ± 35.3	**0.025**

*Male, n*	*72*	*128*		*100*	*40*	
Age	46.7 ± 11.8	51.1 ± 17.0	**0.033**	46.1 ± 9.6	49.5 ± 10.7	0.088
FPG	100.4 ± 30.4	98.2 ± 22.9	0.583	94.8 ± 24.9	96.1 ± 14.4	0.701
BMI	22.4 ± 1.7	20.8 ± 2.3	**< 0.001**	28.9 ± 3.7	28.2 ± 2.2	0.212
TG^α^	140.2 ± 60.0	127.6 ± 54.3	0.125	176.5 ± 75.2	169.1 ± 62.4	0.735
HDL-C^α^	50.1 ± 10.3	49.8 ± 11.2	0.850	44.7 ± 9.1	47.1 ± 7.7	0.113
LDL-C	123.2 ± 34.7	118.3 ± 29.3	0.487	122.2 ± 31.6	131.9 ± 33.8	0.126
TC	201.4 ± 37.9	191.5 ± 34.7	0.068	202.2 ± 36.6	216.6 ± 36.0	**0.039**

*Female, n*	*64*	*122*		*42*	*35*	
Age	45.4 ± 9.8	53.6 ± 16.5	**< 0.001**	46.7 ± 8.5	48.6 ± 12.6	0.452
FPG	89.3 ± 16.1	96.1 ± 13.6	**0.005**	112.2 ± 69.5	93.6 ± 7.9	0.094
BMI	22.4 ± 2.2	20.6 ± 2.9	**< 0.001**	28.1 ± 3.0	27.6 ± 1.9	0.364
TG^α^	110.8 ± 64.6	108.9 ± 51.7	0.500	121.2 ± 57.6	142.2 ± 75.7	0.265
HDL-C^α^	56.7 ± 11.7	58.7 ± 15.1	0.472	56.3 ± 9.2	55.5 ± 10.2	0.612
LDL-C	115.6 ± 33.7	123.0 ± 29.8	0.139	112.7 ± 30.6	121.0 ± 35.7	0.279
TC	194.4 ± 38.5	201.3 ± 34.3	0.231	193.1 ± 34.4	204.9 ± 33.9	0.137

Criteria: Obese BMI ≥25 kg/m^2^[34], High FPG (≥126 mg/dL) [36], high TG (>200 mg/dL), low HDL-C (<40 mg/dL), high LDL-C (≥ 160 mg/dL), high TC (≥240 mg/dL) [37]. Data were presented as means ± SD. Statistical comparisons were calculated between the urban and rural populations in each category, continuous variables were compared using the Welch’s *t*-test, ^α^ Due to skew data distribution, comparisons of TG and HDL were calculated using Mann–Whitney *U*-test. Significant *p* values were in bold.

### Genotypic and allelic distributions

The genotype, minor allele (MAF), and heterozygosity frequencies were moderately equal between urban and rural subjects for all SNPs, both in non-obese and obese group, except for the Ala55Val polymorphism of the non-obese subjects – particularly in males (Table 2). All of the observed genotype frequencies were in Hardy-Weinberg equilibrium (*p* = 0.114 for G(−866)A and *p* = 0.477 for Ala55Val in all; *p* = 0.711 for G(−866)A and *p* = 0.681 for Ala55Val in urban; *p* = 0.091 for G(−866)A and *p* = 0.240 for Ala55Val in rural). The G(−866)A (rs659366) and Ala55Val (rs660339) showed strong linkage disequilibrium with D’ = 0.946 (0.914 for urban and 0.969 for rural subjects) and r^2^ = 0.657 (0.558 for urban and 0.743 for rural subjects).

**Table 2:** Genotypic and allelic distributions of rs659366 and rs659366 in non-obese and obese subjects

	**Variables**	**All**			**Male**			**Female**		
		**Urban**	**Rural**	***p***	**Urban**	**Rural**	***p***	**Urban**	**Rural**	***p***
Non Obese	*n*	*136*	*250*		72	128		64	122	
	**G(−866)A (rs659366)**									
	G/G	0.40	0.29	0.065	0.36	0.23	0.055	0.45	0.34	0.177
	G/A	0.46	0.53		0.54	0.56		0.36	0.50	
	A/A	0.14	0.18		0.10	0.20		0.19	0.16	
	MAF (−866)A	0.37	0.45		0.37	0.48		0.37	0.41	
	Heterozygosity	0.47	0.50		0.47	0.50		0.47	0.48	
	
	**A55V (rs660339)**									
	C/C	0.53	0.39	**0.033**	0.50	0.34	**0.029**	0.56	0.44	0.265
	C/T	0.36	0.43		0.42	0.45		0.30	0.41	
	T/T	0.11	0.18		0.08	0.20		0.14	0.15	
	MAF 55Val	0.29	0.39		0.29	0.43		0.29	0.35	
	Heterozygosity	0.41	0.48		0.42	0.49		0.41	0.46	
Obese	*n*	*142*	*75*		*100*	*40*		*42*	*35*	
	**G(−866)A (rs659366)**									
	G/G	0.27	0.29	0.585	0.28	0.28	0.968	0.24	0.31	0.151
	G/A	0.54	0.57		0.54	0.52		0.55	0.63	
	A/A	0.19	0.13		0.18	0.20		0.21	0.06	
	MAF (−866)A	0.46	0.42		0.45	0.46		0.49	0.37	
	Heterozygosity	0.50	0.49		0.50	0.50		0.51	0.47	
	
	**A55V (rs660339)**									
	C/C	0.38	0.41	0.513	0.40	0.40	0.227	0.33	0.43	0.685
	C/T	0.54	0.47		0.52	0.42		0.57	0.51	
	T/T	0.08	0.12		0.08	0.18		0.10	0.06	
	MAF 55Val	0.35	0.35		0.34	0.39		0.38	0.31	
	Heterozygosity	0.46	0.46		0.45	0.48		0.48	0.44	

Obese criteria: BMI ≥25 kg/m^2^[34]. Statistical comparisons were calculated between the urban and rural populations in each category: genetic profiles were compared using Fisher’s exact test on 2x3 contingency table. Significant *p* values were in bold.

### Association of genotypes with disease traits

Logistic regressions analyses were performed to test the association of the genotypes of each SNPs with high BMI and increasing levels of FPG, TG, LDL-C, and TC, as well as decreasing level of HDL-C (Table 3). Although we did not find any association between genotypes and disease traits after the analyses were adjusted for age, gender, and environment (urban/rural), in a combined analysis of genotypes and environment, the urban subjects carrying the A/A genotype of G(−866)A possessed higher BMI than the rural subjects carrying the same genotype.

**Table 3:** Association of genotypes with disease traits

**G(−866)A (rs659366)**												
**Genotype -Factor**	**BMI**		**FPG**		**TG**		**HDL-C**		**LDL-C**		**TC**	
	**Est.**	***p***	**Est.**	***p***	**Est.**	***p***	**Est.**	***p***	**Est.**	***p***	**Est.**	***p***
G/A	−0.03	0.934	0.28	0.473	0.44	0.157	−0.39	0.136	−0.29	0.220	−0.09	0.702
A/A	−0.44	0.305	0.16	0.755	0.26	0.521	−0.36	0.309	−0.31	0.308	−0.21	0.521
Age	−0.01	0.245	0.04	**<0.001**	−0.01	0.084	0.01	0.141	0.01	**0.009**	0.02	**<0.001**
Male	0.44	**0.016**	0.28	0.284	0.91	**<0.001**	−1.44	**<0.001**	0.24	0.117	0.20	0.211
Urban	0.69	**0.035**	0.12	0.806	0.436	0.204	−0.50	0.103	0.06	0.832	0.07	0.795
G/A-urban	0.57	0.161	−0.11	0.853	−0.11	0.782	0.34	0.377	−0.33	0.333	−0.25	0.480
A/A-urban	1.20	**0.035**	0.58	0.438	−0.28	0.618	0.61	0.236	0.18	0.682	0.23	0.625
**Ala55Val (rs660339)**												
**Genotype -Factor**	**BMI**		**FPG**		**TG**		**HDL-C**		**LDL-C**		**TC**	
	**Est.**	***p***	**Est.**	***p***	**Est.**	***p***	**Est.**	***p***	**Est.**	***p***	**Est.**	***p***
C/T	−0.05	0.859	−0.11	0.756	0.46	0.123	−0.19	0.446	−0.11	0.629	0.08	0.737
T/T	−0.55	0.195	−0.03	0.945	0.26	0.488	−0.08	0.811	−0.25	0.391	−0.11	0.741
Age	−0.01	0.222	0.04	**<0.001**	−0.01	0.103	0.01	0.133	0.02	**0.007**	0.02	**<0.001**
Male	0.43	**0.019**	0.30	0.254	0.90	**<0.001**	−1.45	**<0.001**	0.21	0.158	0.19	0.249
Urban	0.73	**0.010**	0.04	0.787	0.46	0.113	−0.30	0.268	−0.09	0.701	0.02	0.931
C/T-urban	0.77	**0.046**	−0.04	0.945	−0.12	0.760	0.11	0.771	−0.04	0.914	−0.09	0.787
T/T-urban	0.70	0.253	0.57	0.452	−0.87	0.167	0.35	0.528	0.35	0.469	0.11	0.826

Logistic regression analyses of genotypes association with disease traits, adjusted for age, gender, and population (urban/rural). Criteria: Obesity (BMI ≥25 kg/m^2^) [34], high FPG (≥126 mg/dL) [36], high TG (200–499 mg/dL), low HDL-C (<40 mg/dL), high LDL-C (≥ 160 mg/dL), high TC (≥240 mg/dL) [37]. Significant *p* values obtained from Wald test were in bold.

For the Ala55Val polymorphism, there was no association of all genotypes (C/C, C/T, or T/T) with disease traits, but more urban than rural subjects with C/T genotype (Ala55Val) has higher BMI. Although not statistically significant, the urban subjects with T/T genotype correspondingly showed a tendency for having a higher BMI than their rural counterparts, marked by high estimation value of 0.70.

Despite the nonexistent trend in genotypes, we found that increasing level of FPG, LDL-C and TC was influenced by age. While HDL-C level was lower, BMI and TG level was higher in male than female subjects. Consistent with our previous observation in non-obese subjects, we found that urban subjects have higher BMI than rural subjects, suggesting the influence of environmental factors.

### Association of UCP2 haplotypes with disease traits

Since the two SNPs studied were in strong linkage disequilibrium (D’ = 0.946 and r^2^ = 0.657), we therefore conducted logistic regressions to assess the association between haplotypes and the disease traits adjusted for the subject’s age, gender and environment (urban/rural). Frequencies of all possible haplotypes were determined by the expectation maximization (EM) algorithm, and only haplotypes with a frequency above 5% were taken for further analysis. The haplotype consisting both of the major allele GC ((−866)G-55Ala) was the most common (56%), followed by the haplotype consisting both of the minor allele AT ((−866)A-55Val) (34%), and AC ((−866)A-55Ala) (8%). The frequency of haplotype GT ((−866)G-55Ala) was only 1%, thus excluded from the analysis.

In Table 4, we presented the analyses for the additive and dominant genetic models, except the recessive model that not feasible to be analyzed due to the low frequency of recessive haplotype GT. The general outcomes of the additive and dominant models were in parallel. There was no association of any haplotype with the diseases traits, except when influenced with the urban environment, as indicated by high BMI in urban subjects carrying the AT haplotype (*p*-value in additive/dominant model = 0.032/0.024). However, subjects with the AC haplotype showed a tendency for high FPG (estimate values of 0.82 for the additive model, and 0.77 for the dominant model, respectively). A noticeable trend of high TG (estimate values of 0.98 for the additive model, 1.04 for the dominant model) and low HDL-C estimate values of 0.97 for the additive model, and 0.89 for the dominant model) in urban subjects carrying the AC haplotype was observed, although both models did not produce any significant haplotype-environment interaction.

**Table 4:** Association of UCP2 haplotypes with disease traits

**Additive Model**													
**Haplotype**	**f**	**High BMI**		**High FPG**		**High TG**		**Low HDL-C**		**High LDL-C**		**High TC**	
		**Est.**	***p***	**Est.**	***p***	**Est.**	***p***	**Est.**	***p***	**Est.**	***p***	**Est.**	***p***
GC	0.56												
AC	0.09	−0.05	0.902	0.82	0.280	−0.23	0.696	−0.58	0.325	−0.48	0.461	−0.65	0.315
AT	0.34	−0.21	0.312	0.33	0.444	0.11	0.676	−0.37	0.211	0.00	0.996	0.21	0.379
GT	0.01	NA	NA	NA	NA	NA	NA	NA	NA	NA	NA	NA	NA
Age		−0.01	0.222	0.05	**<0.001**	−0.02	0.109	0.01	0.335	0.02	0.086	0.01	**0.024**
Male		0.46	**0.013**	0.55	0.155	0.80	**0.002**	−1.89	**<0.001**	0.22	0.419	0.25	0.310
Urban		0.74	**0.013**	0.89	0.218	0.10	0.805	0.90	0.053	0.62	0.135	0.46	0.247
AC-urban		0.24	0.643	0.19	0.835	0.98	0.160	0.97	0.191	−0.44	0.612	−0.04	0.960
AT-urban		0.61	**0.032**	0.18	0.745	−0.04	0.912	0.49	0.228	−0.40	0.329	−0.42	0.261
GT-urban		NA	NA	NA	NA	NA	NA	NA	NA	NA	NA	NA	NA
**Dominant Model**													
**Haplotype**	**f**	**High BMI**		**High FPG**		**High TG**		**Low HDL-C**		**High LDL-C**		**High TC**	
		**Est.**	***p***	**Est.**	***p***	**Est.**	***p***	**Est.**	***p***	**Est.**	***p***	**Est.**	***p***
GC	0.56												
AC	0.09	0.05	0.900	0.77	0.341	−0.19	0.761	−0.60	0.336	−0.49	0.465	−0.64	0.337
AT	0.34	−0.14	0.644	0.26	0.701	0.18	0.644	−0.50	0.303	−0.05	0.882	0.28	0.450
GT	0.01	NA	NA	NA	NA	NA	NA	NA	NA	NA	NA	NA	NA
Age		−0.01	0.234	0.05	**<0.001**	−0.01	0.112	0.01	0.323	0.02	0.100	0.02	**0.025**
Male		0.46	**0.017**	0.53	0.168	0.80	**0.002**	−1.89	**<0.001**	0.22	0.403	0.25	0.309
Urban		0.67	**0.038**	1.04	0.155	−0.08	0.852	0.72	0.171	0.54	0.228	0.41	0.341
AC-urban		0.18	0.739	0.09	0.927	1.04	0.146	0.89	0.247	−0.38	0.668	0.00	1.000
AT-urban		0.89	**0.024**	−0.01	0.987	0.24	0.637	0.34	0.577	−0.33	0.548	−0.46	0.383
GT-urban		NA	NA	NA	NA	NA	NA	NA	NA	NA	NA	NA	NA

Logistic regression analysis for association of UCP2 haplotypes with diseases traits, assuming the additive and dominant genetic models, adjusted for age, gender, and population (urban/rural). Significant *p* values from Wald test were in bold. Analysis for a recessive model was not feasible due to low frequency of rare haplotype (<5%). Criteria: Obesity (high BMI ≥25 kg/m^2^) [34], High FPG (≥126 mg/dL) [36], high TG (200–499 mg/dL), low HDL-C (<40 mg/dL), high LDL-C (≥ 160 mg/dL), high TC (≥240 mg/dL) [37]. f: haplotype frequencies. NA: not available, data were omitted due to low haplotype frequencies (<5%).

Independent from any haplotype, high FPG was affected by age, and the male subjects were subjected to increased tendency for high BMI, high TG and low HDL-C. Similarly observed in our genotype analyses, high BMI was induced by urban environment.

## Discussion

In the present study of the Balinese population, we examined the association of two commonly observed UCP2 gene polymorphisms, the G(−866)A in the promoter region (rs659366) and the Ala55Val in exon 4 (rs660339), with obesity, high FPG and high serum lipids. Uncoupling protein 2 has been reported to play an important role in the regulation of human energy metabolism, through its functions of proton gradient dissipation, and uncoupling of respiration from oxidative phosphorylation that consequently converted ATP to heat. Thus, variations in the UCP2 gene was proposed as potential modulator of energy balance.

### Urban lifestyle affected BMI

This study provided evidence concerning the role of UCP 2 gene polymorphisms in influencing the increasing prevalence of obesity. Moreover, this study has the advantage of being conducted in a relatively homogeneous population in terms of genetic backgrounds, considering Bali is a small island, and intermarriage between Balinese with individuals from other ethnic groups is still relatively uncommon. Also, as a population that has undergone swift lifestyle changes for the last 20 years due to rapid developments in the tourism industry, the traditional Balinese has become more sedentary [28,32,33]. The effect of these lifestyle changes is reflected by the high prevalence of obesity in the urban as compared to the rural Balinese. Furthermore, BMI was higher in the urban subjects (both male and female) of the non-obese group, showing that lifestyle changes influenced the increasing BMI.

In spite of the fact that this study has a power limitation for the genetic analyses due to the relatively small sample size, in particular in the sub-group analyses, we found that the MAFs for both G(−866)A and Ala55Val SNPs of the UCP2 genes were rather high, showing that these polymorphisms were relatively frequent in this population.

### UCP2 G(−866)A influenced the increased of BMI

In the Balinese population studied, we observed that urban subjects with A/A genotype of the SNP G(−866)A has higher BMI than rural subjects with the same genotype, suggesting the important role of the environment. Several studies have reported that the A-allele or AA genotype is associated with obesity [22,45]. In a study conducted in Korean women undergoing a very low-calorie program, the A/A genotype of this SNP lost less weight than carriers of the G-allele [46]. Two other studies reported that in obese children with the A/A genotype the resting energy expenditure was increased [47], and in Pima Indians the SNP was associated with increased 24-hour energy expenditure [48]. A recent study reported a strong effect of sibutramine on weight and body fat percentage loss in the combined UCP2 A/A + G/A of the SNP G(−866)A in a Taiwanese population [49]. Interesting to note that in their placebo group, the A/A genotype showed the least weight and fat percentage loss, which was in agreement with a study conducted in Korean women [46], indicating that the effect of the UCP2 G(−866)A polymorphism might be universal, at least for Asian.

Even though marginal, there was also an association of the Balinese with C/T genotype of the Ala55Val SNP with high BMI when influenced by the urban environment, but we failed to detect the same association with subjects possessing the homozygous risk alleles T/T, which indicates that the interaction of UCP2 genotypes and environment is not straightforward.

Although we did not observe any significant association with other traits, a previous study has reported that subjects with the (−866)A/A genotype had significantly reduced LDL particle size, a significant predictor of the development for coronary artery disease, than those with the (−866)G/G genotype [26]. Several studies have confirmed the association of either G(−866)A and/or Ala55Val variants with T2DM [18,23,25,27,31]. In the obese state, the risk would be increased due to insulin resistance, which may accompany obesity and impairment of glucose-stimulated insulin secretion attributable to UCP2 at-risk genotype [50]. Excess of fat storage in the obesity state may lead to local insulin resistance, and might stimulate a chronic, sub-acute state of inflammation. A study reported an association of the high-sensitivity C reactive protein (hs-CRP), a biomarker of inflammation that is also a powerful risk marker for cardiovascular disease, with the G(−866)A polymorphism, thus suggesting the role of UCP2 gene in the regulation of the inflammatory response [51]. The lack of association of individual UCP2 SNPs and disease traits in the Balinese population might be due to the influence of other genetic or environmental factors that were not studied in this research, such as the MTNR1B gene polymorphism that has been reported to be associated with type 2 diabetes mellitus and lipid levels in Han Chinese population [52].

### UCP2 haplotypes interacted with environment

The G(−866)A and Ala55Val SNPs were in strong linkage disequilibrium. Therefore, the effect of one SNP cannot be separated from the other. To address the influence of combined UCP2 variants on high BMI, high levels of FPG, TG, LDL-C, and TC, as well as low level of HDL-C, we generated UCP2 haplotypes and tested the association between haplotypes and disease traits. Again, we did not find significant association between haplotypes and traits, unless influenced by the urban environment. The AT ((−866)A-55Val) haplotype showed an association with high BMI in urban, in both additive and dominant models. A trend of high FPG was observed in carriers of AC ((−866)A-55Ala) haplotype. We also noticed a tendencies of high TG, and low HDL-C in urban subjects carrying the AC ((−866)A-55Ala) haplotype, although not significant for haplotype-environment interaction in both models, which might be due to the low frequency of this haplotype in the population studied.

In our study, the level of FPG and TC in the Balinese increased with age, and male subjects have more tendency for high BMI than female subjects. A study conducted in non-diabetic Chinese reported a 0.15 mmol/L increase in FPG for every decade of increase in age [53]. In a previous study to determine the effects of sex and ovarian hormones carried out in mice, it was reported that male mice had a greater susceptibility of body weight gain than female mice [54].

In this study, we did not find significant association between UCP2 gene polymorphisms and disease traits. However, we were able to demonstrate that in an urban environment, the A/A genotype of the G(−866)A SNP and the AT haplotype were associated with high BMI, and consequently resulted in high prevalence of obesity. Discrepancies between our results and others might be due not only to different ethnic groups and genetic makeup, but also to the influence of various lifestyle and environmental exposures, as well as the responses to these exposures. Thus, the role of the UCP2 variants as obesity and/or diabetes risk factor might be overshadowed by environmental influence in some populations. Nonetheless, this study and others have emphasized the important role of UCP2 in metabolism, and therefore further investigations are needed to confirm its specific role.

## Conclusions

Our study has provided compelling evidence regarding the importance of environmental settings in studying the involvement of UCP2 gene polymorphisms in the development of obesity in a Balinese population. We demonstrated that the UCP2 gene polymorphisms G(−866)A and Ala55Val are associated with obesity in Balinese, but only if the urban environment was taken into account. However, the above interaction appeared nonexclusive, since other genetic or environmental factors that were not studied in this research might modify the outcome. Therefore, it is imperative to conduct further studies using more genetic markers.

## Abbreviations

SNP: Single Nucleotide Polymorphism; UCP2: Uncoupling protein 2; FPG: Fasting plasma glucose; BMI: Body mass index; TG: Triglyceride; HDL-C: High density lipoprotein cholesterol; LDL-C: Low density lipoprotein cholesterol; TC: Total cholesterol; MAF: Minor allele frequency; T2DM: Type 2 Diabetes Mellitus.

## Competing interest

The authors declare that they have no competing interests.

## Authors’ contributions

SO carried out molecular genetic studies and statistical analysis, and drafted the manuscript, HT performed statistical analysis and interpretation of the data, KS and HS participated in the design of the study, provided direction, and helped revise the final manuscript, MRS and PD coordinated volunteers recruitment, performed clinical assessments and data analysis, WA and CAF carried out some of the genetic analysis, and helped revise the manuscript, SGM designed the study, performed data analysis and interpretation of the data, provided direction, and revised the final manuscript. All authors read and approved the final manuscript.
